# Microwave technology for detecting traumatic intracranial bleedings: tests on phantom of subdural hematoma and numerical simulations

**DOI:** 10.1007/s11517-016-1578-6

**Published:** 2016-10-13

**Authors:** Stefan Candefjord, Johan Winges, Ahzaz Ahmad Malik, Yinan Yu, Thomas Rylander, Tomas McKelvey, Andreas Fhager, Mikael Elam, Mikael Persson

**Affiliations:** 1 Department of Signals and Systems, Chalmers University of Technology, 412 96 Gothenburg, Sweden; 2 MedTech West, Sahlgrenska University Hospital, Röda Stråket 10 B, 413 45 Gothenburg, Sweden; 3 SAFER Vehicle and Traffic Safety Centre at Chalmers, Gothenburg, Sweden; 4 Clinical Neurophysiology, Sahlgrenska University Hospital, Blå Stråket 5, 413 45 Gothenburg, Sweden

**Keywords:** Traumatic brain injury, Intracranial bleedings, Microwave technology, Subdural hematoma phantom, Finite element method

## Abstract

Traumatic brain injury is the leading cause of death and severe disability for young people and a major public health problem for elderly. Many patients with intracranial bleeding are treated too late, because they initially show no symptoms of severe injury and are not transported to a trauma center. There is a need for a method to detect intracranial bleedings in the prehospital setting. In this study, we investigate whether broadband microwave technology (MWT) in conjunction with a diagnostic algorithm can detect subdural hematoma (SDH). A human cranium phantom and numerical simulations of SDH are used. Four phantoms with SDH 0, 40, 70 and 110 mL are measured with a MWT instrument. The simulated dataset consists of 1500 observations. Classification accuracy is assessed using fivefold cross-validation, and a validation dataset never used for training. The total accuracy is 100 and 82–96 % for phantom measurements and simulated data, respectively. Sensitivity and specificity for bleeding detection were 100 and 96 %, respectively, for the simulated data. SDH of different sizes is differentiated. The classifier requires training dataset size in order of 150 observations per class to achieve high accuracy. We conclude that the results indicate that MWT can detect and estimate the size of SDH. This is promising for developing MWT to be used for prehospital diagnosis of intracranial bleedings.

## Introduction

Traumatic brain injury (TBI) is termed “the silent epidemic.” It strikes up to 2 % of the population each year and is the leading cause of death and severe disability among young people [3, 10]. Furthermore, it affects many elderly, who are more vulnerable due to widespread use of anticoagulant medications and lower brain plasticity [31, 33]. Road traffic injury, sports injuries, assaults and combat operations commonly produce TBI, and elderly are often exposed to accidental falls [3, 6, 10, 33]. Intracranial bleedings constitute the most important complication of TBI [3]. Larger bleedings must be evacuated promptly to save the lives of these patients and mitigate injury [3, 34]. Unfortunately, many patients are treated too late [4, 16, 34]. Patients without clear symptoms, such as decreased level of consciousness, are often undertriaged in the prehospital phase [13]. Delay of treatment due to patients initially being transported to a non-trauma center, to be later transferred to a trauma center, causes substantial mortality [13, 16]. Therefore, there is a need for methods for the prehospital setting to predict the need for lifesaving interventions from clinical observations, as demonstrated by Liu et al. [18] using vital sign measurements and a machine-learning algorithm, and to directly detect occult injury such as intracranial bleedings.

The clinical standard for detecting intracranial bleedings is computed tomography (CT) [10]. The main disadvantage with CT is that it is not well suited for field use. Although mobile units with CT have been developed for examining stroke patients [35], it would be advantageous to incorporate devices for detecting intracranial bleedings in ordinary road and air ambulances. One candidate is a recently developed handheld instrument using near-infrared spectroscopy, the Infrascanner [17, 28, 30]. Its principle for detection is to compare the amount of light absorption for the left and right brain hemispheres, by four point measurements on opposite sides of the skull. Strong asymmetry is likely due to a hematoma on the side with largest absorption, since extravascular blood contains tenfold the concentration of hemoglobin compared to intravascular blood [17]. In a multicenter study including 365 patients, of whom 96 were confirmed to have intracranial hemorrhage, detection sensitivity of 88 % and specificity of 91 % were shown [28]. This result applied for bleedings larger than 3.5 mL, closer than 2.5 cm from the surface of the brain. If all bleedings were included, the sensitivity decreased to 69 % [28]. The disadvantages with the Infrascanner are that it cannot give detailed information about location and size of hematoma [28]. It cannot detect bleedings deep inside the brain, such as deep-sited intraparenchymal or intraventricular hemorrhage, due to the limited penetration depth of approximately 2.5 cm [28]. Another possible future candidate is electrical impedance tomography. Oh et al. [24] showed using epidural electrodes placed on exposed cortex of anesthetized rats that somatosensory evoked responses could be recorded with an SNR of >50 at 225 Hz. That study could pave the way for imaging fast neural activity in the brain, which could be used for detecting and monitoring TBI [24]. However, a shortcoming of electrical impedance tomography is that the cranium has electrically insulating properties that limit current penetration and impede deep imaging of the brain without using implanted electrodes [20].

We propose the use of broadband microwave technology (MWT) [27], in conjunction with a diagnostic algorithm, to detect and classify different types of intracranial bleedings. Recently, Persson et al. [26] showed that MWT can distinguish hemorrhagic stroke from ischemic stroke, in the two first proof-of-principle clinical studies. Twenty and 25 patients with acute stroke were included, respectively. The patients were measured with an antenna system worn on the head. The article demonstrated areas under the receiver operating characteristic curves of 0.85 and 0.88, respectively, using a machine-learning algorithm based on singular value decomposition (SVD). At 90 % detection sensitivity for hemorrhagic stroke, the specificity was 65 %, for the second clinical study [26].

MWT can detect bleedings due to the dielectric contrast between blood and brain matter [9, 26]. MWT could suite for field use since cheap, fast and portable systems working in the time domain are emerging [26, 27, 38]. An advantage with MWT is that it employs low-power ($\sim$1 mW) microwave signals. MWT has the potential to detect lesions deep inside the brain, since microwaves will penetrate the skull and brain, and transmission signals between antennas on opposite sides of the head can be utilized [26]. Furthermore, a patient could potentially be monitored in real time, to follow-up on the progression of injury. Such monitoring is not appropriate with CT, i.e., due to excessive ionizing radiation. Our long-term goal is to incorporate MWT into road and air ambulances, to increase the precision of triage and decrease the time to treatment for trauma and stroke patients.

In the future, it may also be possible to image the brain and visualize stroke and TBI lesions [11, 12, 14, 19, 21, 22, 32, 36, 37]. Contemporary systems do not provide detailed anatomical images compared to CT, but may still be valuable for detection of intracranial hemorrhage, see, e.g., [21, 22]. Current imaging algorithms are not sufficiently fast to enable prehospital use without use of a priori information [19]. Mobashsher et al. [21] showed that a portable wideband microwave system with a custom unidirectional antenna has potential to visualize intracranial bleedings. They used a realistic phantom fabricated by a 3D printer. The research group has presented several further studies on improvements of image reconstruction to achieve fast and accurate imaging [11, 12, 36, 37]. Recently, Mahmood et al. [19] showed that a priori information from magnetic resonance imaging and automatic segmentation of tissue types can be used to facilitate rapid imaging of intracranial bleedings.

The advantage of a machine-learning approach as compared to imaging is that a classification algorithm can generate a diagnosis in real time without need for physician/operator image interpretation. The disadvantages are that not as detailed information about the lesion is presented, and that an accurate classifier may require a large set of training data. We have shown that MWT has potential to localize traumatic intracranial bleedings using a diagnostic algorithm [5]. That study focused fully on localization of bleedings. It did not evaluate detection accuracy.

In this study, we assess the potential of MWT for detecting traumatic intracranial bleedings, using a microwave helmet and a classification algorithm based on SVD. We focus on subdural hematoma (SDH), which has very high mortality: 50–85 % [10]. It is the traumatic intracranial bleeding that most commonly requires surgical evacuation [10]. We model SDH by constructing a phantom in a human cranium. Furthermore, we perform numerical simulations to mimic expected inter-variability from microwave measurements on an SDH patient population, by modeling SDH of several different sizes, at different locations, in crania of varying sizes, with a layer of cerebrospinal fluid (CSF) of varying thickness.

## Methods

### Phantom construction

**Fig. 1:**
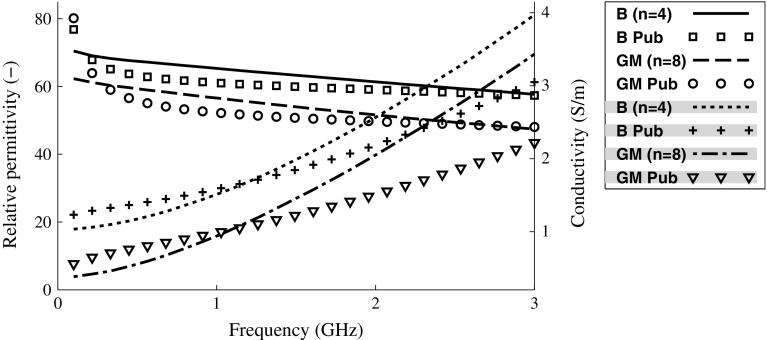
Measurements of dielectric properties of phantom solutions compared to published values [1]. The shading in the legend box denotes traces belonging to the right-hand side axis. *B* blood, *GM* gray matter, *Pub* published values. The mean of measurements on several phantom solutions is shown (*n* denotes number). Standard deviations were small compared to the differences between blood and gray matter; they have been omitted for clarity

Solutions for mimicking the dielectric properties of blood and gray brain matter were created by mixing water, sugar, agar (Agar fine powder, Sigma-Aldrich, St. Louis, USA) and salt, in proportions according to Table 1. The agar was used to make the solutions solidify into a relatively stiff gel. Formaldehyde (Formaldehyde solution 37 wt% H$_2$O, Sigma-Aldrich) was added, 3 mL per L of phantom solution, to protect the phantoms from mold. Small quantity of red color (Röd hushållsfärg, Dr. Oetker Sverige AB, Mölndal, Sweden) was added to the bleeding phantom. Prior to adding agar, the dielectric properties were measured using a network analyzer (PNA, Agilent E8362B, Agilent Technologies, Santa Clara, CA, USA) and a dielectric probe kit (Agilent 85070E). They were similar to published values [8, 9], see Fig. 1. Measurements of dielectric properties were done before adding agar, because it was easier to avoid air between the probe tip and the measurement object when the solutions were in liquid state. The dielectric properties will not change substantially by adding agar [29]. After adding agar, the solutions were heated to 70–80 $^{\circ }\hbox {C}$. They were kept at that temperature for 4–5 min while stirring.

**Table 1:** Recipes for blood and gray matter phantoms (volume percent)

Ingredients	Blood (%)	Gray matter (%)
Water (deionized)	67.8	57.9
Sugar	26	36
Agar	6	6
Salt	0.2	0.1

**Fig. 2:**
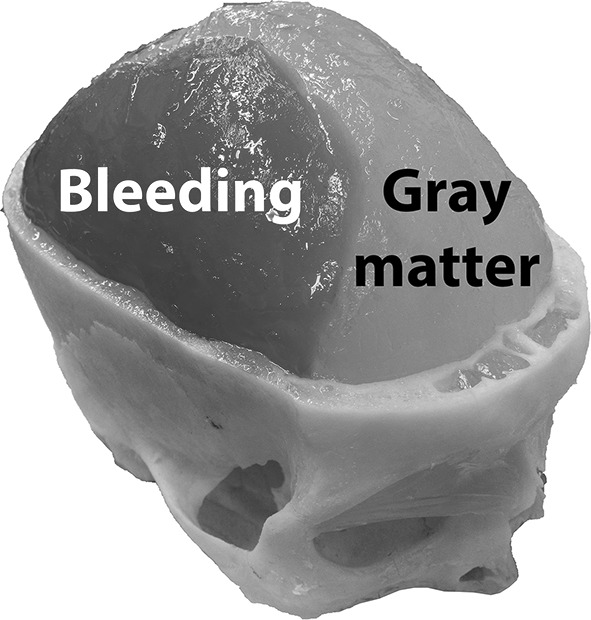
Model of SDH of approximate size 110 mL. The upper part of the skull was removed when the photograph was taken

A human cranium that was divided at the upper part of the skull was used to contain the brain phantom. The cranium was sealed by tape. The liquid brain matter solution was poured into the cranium via foramen magnum. After the solution had solidified, the upper part of the skull was removed and the integrity of the brain phantom was verified. The cranium was then sealed again, and measurements were performed (explained in Sect. 2.2), which were used as reference for simulating the case of a healthy patient, i.e., without any bleeding. Afterward, a model of SDH was constructed by cutting away a crescent-shaped [3] portion of the brain phantom with a scalpel and refill with blood phantom solution, see Fig. 2. The blood phantom solution was poured into place when it was in a semisolid state, so it filled out the cutaway part completely and the surface could be molded to follow the shape of the brain’s surface. It was then allowed to solidify completely. After measurements, the bleeding was cut away and a larger bleeding was constructed. This was repeated for each new bleeding size. In total, four phantoms were created using the same brain phantom as base, one without bleeding and three with bleeding sizes of approximately 40 mL (thickness 0.5 cm), 70 mL (thickness 1 cm) and 110 mL (thickness 1.5 cm). The aim was to create realistic shapes and sizes. Focus was on obtaining target thickness (the maximum distance from the inner surface of the cranium to the edge of the bleeding). The thickness was measured with a ruler. The sizes corresponded to clinically relevant SDH of different degrees of severity. Surgical evacuation is recommended if the SDH is thicker than 1 cm [3].

### Phantom measurements

**Fig. 3:**
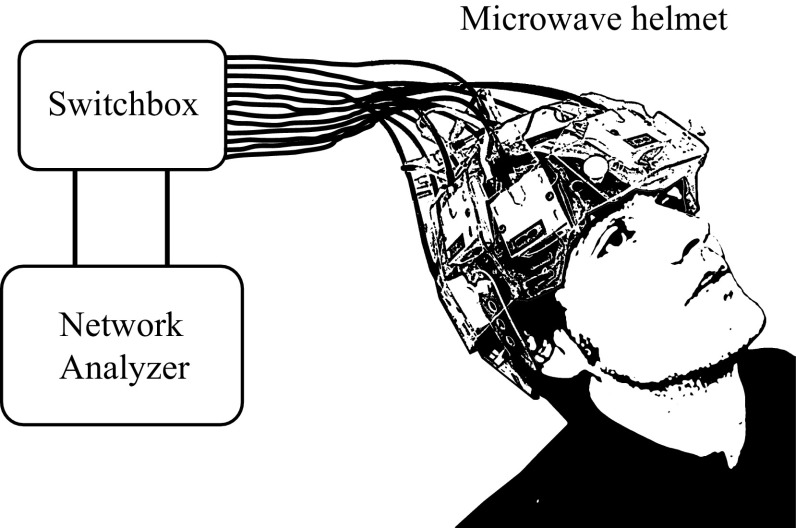
Schematic drawing of the experimental setup

A microwave helmet with 12 broadband antennas [27] was connected to a network analyzer with a built-in switching matrix (Strokefinder R10, Medfield Diagnostics AB, Gothenburg, Sweden), see Fig. 3. To ensure good electromagnetic coupling to the measurement object, water bags were attached to the antennas using dual-stitching tape. The helmet was hung upside down, the cranium was inserted into the helmet, and the water bags were then filled. Two of the antennas at the neck were not used, since they did not obtain close contact with the (relatively small) cranium. To simulate measurements on patients, where the helmet position will vary, we took the cranium out of the helmet, emptied the water bags and repositioned the cranium in the helmet after every third measurement. Thirty measurements were performed on each phantom, for a total of 120 measurements. The frequency range 0.1–3.0 GHz was measured, at a step size of 7.25 MHz. Each antenna in turn acted as transmitter. The complex amplitude of the scattering parameters $S_{ij}$ was measured, where $S_{ii}$ are reflection coefficients and $S_{ij}$ transmission coefficients, and *i* and *j* are antenna indices. The measurements $j<i$ were disregarded due to reciprocity, $S_{ij} =S_{ji}$. In total, there were 55 channels for the ten antennas. All measurements were performed within 48 h from phantom construction.

### Numerical simulations

**Fig. 4:**
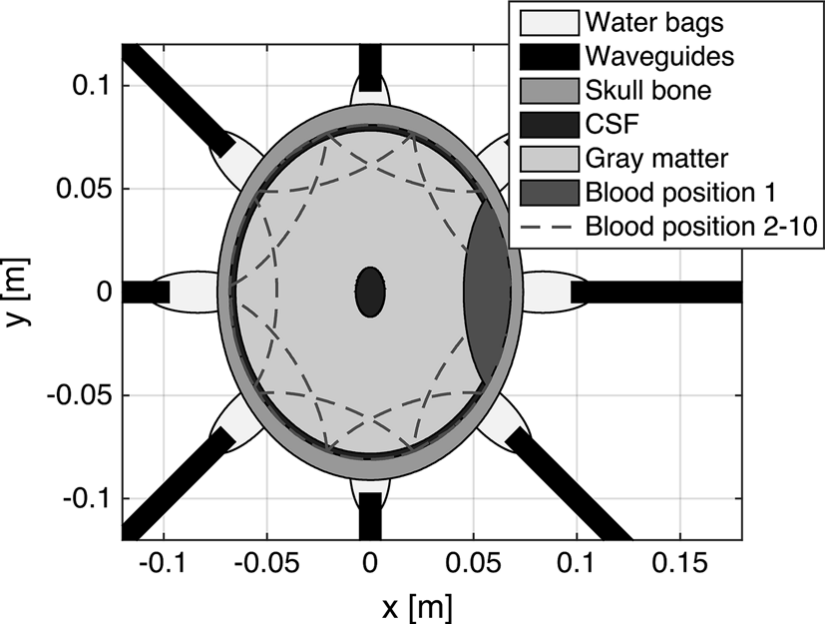
2D simulation model for patient inter-variability robustness investigation. The bleedings depicted have a thickness of 2 cm

To investigate“ness of the classification, with respect to patient inter-variability, a 2D numerical simulation of the measurement setup was performed (Fig. 4). It was based on the finite element method [15] and implemented in MATLAB (version R2012a, MathWorks Inc., Natick, MA, USA). Eight antennas were used, resembling a 2D slice of the measurement setup. To match the broad frequency band excitation for the laboratory measurements, the antennas were modeled as parallel-plate waveguides, filled with a material with relative permittivity 20 (conductivity zero). The water bags connecting the port openings to the skull were introduced in the final model, which resulted in a substantial increase in the power transmitted into the head. We used values for the frequency-dependent dielectric properties of the skull bone and CSF from [1, 8]. Values for gray matter and blood matched the measurements of the phantom. Values for water was calculated using a Debye model  [23]. Five parameters were included in the model:

1. Bleeding size: Crescent-shaped bleedings with thicknesses of 0.2, 0.5, 1, 2 and 2.5 cm.
2. Bleeding positions: Ten positions evenly spaced along the brain–cranium interface. Adjacent positions overlapped for bleedings thicker than 0.5 cm. For practical reasons, we utilized the geometrical symmetry to reduce the total simulation time. Bleedings were first created on the right side of the brain only. Bleedings on the left side were created by reusing the already computed scattering parameters of the right side simulations.
3. Head size: Seven sizes were modeled. The original head was an ellipse of size $18.2 \times {14.8}\,\mathrm{cm}$, equal to the dimensions of the human cranium used for the laboratory phantom. We modeled head sizes of ±6 %, in steps of 2 %, from the original head size.
4. CSF: The thickness of the layer beneath the skull bone was varied randomly between 2 and 5 mm. In addition, a small elliptic CSF region in the interior of the brain was added. This elliptic region was included to coarsely mimic inhomogeneities in the center of the brain that are expected from the ventricular system.
5. Helmet position: The head was rotated randomly between ±6$^{\circ }$ relative to the antenna array. This corresponds to a maximal rotation of the helmet of approximately $\pm 1$ cm relative to a patient’s forehead.

A dataset with 250 observations per bleeding size and 250 observations without bleeding was created, for a total of 1500 observations. Each observation was randomly drawn, without replacement, from a large dataset with approximately even distributions of bleeding sizes, head sizes and helmet positions. Thus, each class of bleedings, i.e., each bleeding size, was composed of many different geometric scenarios, mimicking patient measurements.

To evaluate classifier performance on unseen data, an additional dataset, here referred to as the validation dataset, was created. The same procedure as described above was used but with different parameter values for bleeding size and head size, spanning approximately the same ranges as for the original data, i.e., the dataset described above. The classifier was trained on the original data, and we then tested whether the case of “no bleeding” could be differentiated from bleedings of any size, for the validation dataset.

### Data preprocessing and analysis

The magnitude and phase of the raw reflection $S_{ii}$ and transmission coefficients $S_{ij}$ were plotted for visual comparison between measurements and simulations of SDH of different sizes to no bleeding, to evaluate whether there are any clear visual trends due to the presence/size of SDH compared to a healthy brain.

MATLAB (version R2012a) was used for all preprocessing and analysis. Algorithms included in MATLAB were used when possible, and other algorithms were written in-house.

#### Preprocessing

For each measurement, all frequencies of the reflection $S_{ii}$ and transmission coefficients $S_{ij}$ were combined into one complex vector *x*. As an example, for the phantom measurements the 55 channels, each consisting of 401 frequencies, were combined into a vector with a total of $55 \times 401 = {22{,}055}$ elements. No further preprocessing of the raw data was performed.

#### Classification algorithm

The classification algorithm is based on the same principles as used in [26], but the implementation in this study enables classification of multiple classes. Given *C* the total number of classes, let $\mathscr {U}_c$ be a subset containing data from class *c*, where $c \in \{1,\cdots ,C \}$. Hence, $\forall$ ${x}_{c,i} \in \mathscr {U}_c$ the signal is modeled as:

1 $$\begin{aligned} {x}_{c,i}={U}_c {\alpha }_c(i) + {e}_c \end{aligned}$$

where, ${U}_c$ is the subspace basis with a dimension $m_c$; vector ${\alpha }_c(i)$ contains the weights of the basis vectors, and ${e}_c$ is additive white noise [5, 25]. Therefore, the classification criterion is to find the class *c* with the smallest distance $d_c$ from the data ${x}_i$ to the basis ${U}_c$. The detailed classification algorithm is shown in Algorithm 1. 
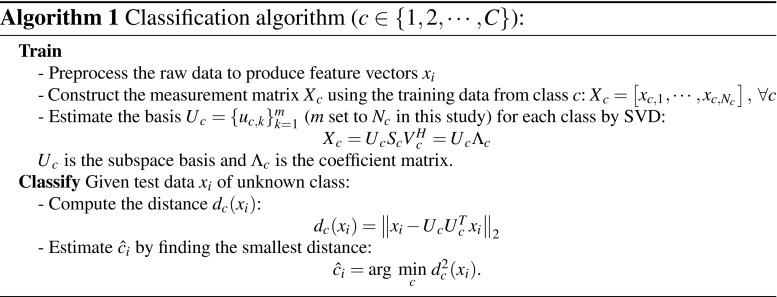


#### Validation

Fivefold cross-validation [2] was used to estimate the classification accuracy for both laboratory and simulated data (except for the validation dataset). This means that the dataset was divided into five randomized folds with approximately equal number of observations. One fold at a time was left out. The classifier was trained on data from the remaining four folds, and the observations in the left out fold were then classified. This procedure was repeated for all folds, i.e., performed five times.

For the laboratory experiments, we considered measurements where the helmet position was unchanged (the cranium was repositioned after every third measurement) to be replicate measurements. They were treated as one unit that was included in either training or validation data, to avoid introducing bias due to replicate measurements being present in training data.

For the simulated data, the classification accuracy as a function of number of samples per class was assessed by extracting random subsets from the full dataset and employing fivefold cross-validation on each subset. This was done to evaluate how large training dataset the classifier needs to achieve high accuracy. The separate validation dataset, with other values of bleeding and head sizes, was used to confirm that our model was not overfitted. The original dataset was used as training data, and the classification accuracy was calculated by dividing the validation data into only two classes: No bleeding and Bleeding. If an observation in the validation dataset was closest to the subspace basis of the No bleeding class of the original data, it was classified as No bleeding, else if closest to any of the bleeding subspaces of the original data, it was classified as Bleeding.

## Results

**Fig. 5:**
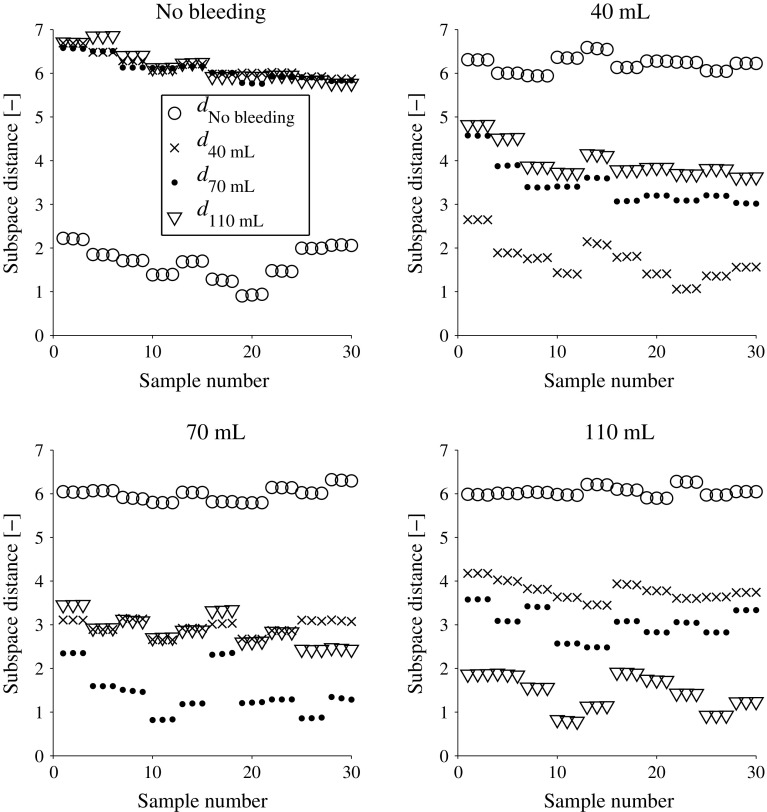
Distances $d_c$ to each subspace for all observations for the laboratory data. The class *c* is given in the plot titles. We see that the classification accuracy is 100 % since all observations have shortest subspace distance to their respective class (1). We also see that there is a tendency that bleedings of sizes close to each other are more similar than bleeding sizes far apart, as measured by the subspace distances, e.g., for the test on 110 mL is $d_{110\,\mathrm {mL}}< d_{70\,\mathrm {mL}}< d_{40\,\mathrm {mL}} < d_{\mathrm {No\;bleeding}}$ (*bottom right plot*). The variability caused by taking the helmet on and off every third measurement shows in the plots; groups with repeated observations are close to each other, whereas there can be a relatively large difference between groups

**Fig. 6:**
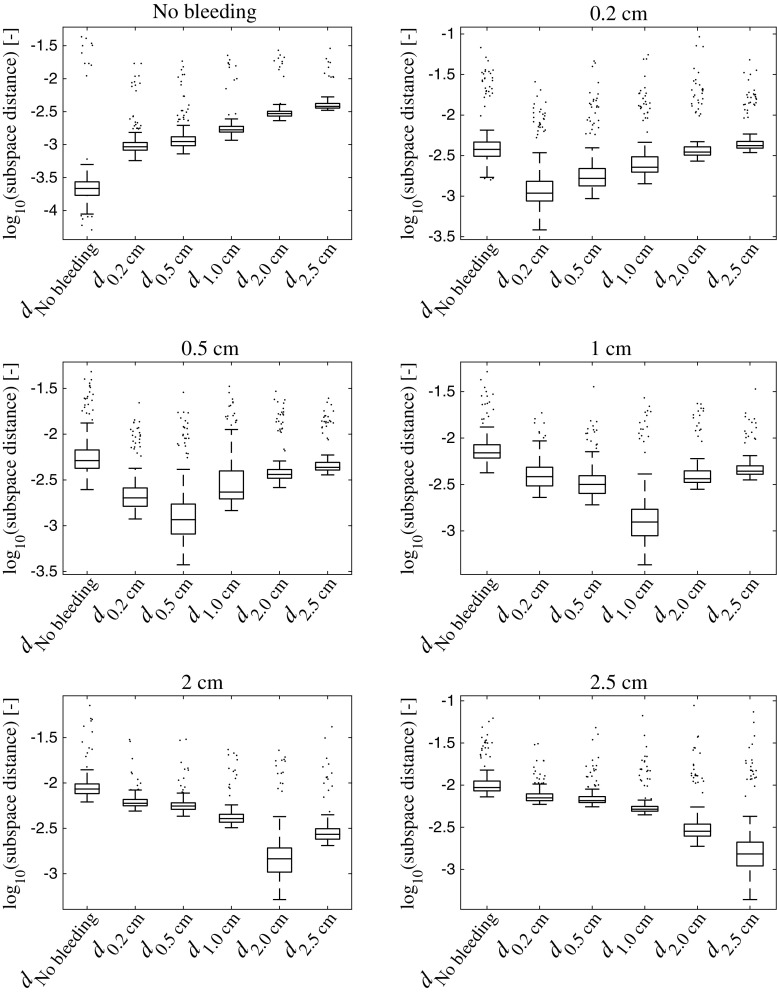
*Box plots* showing the distances $d_c$ to each subspace, for all classes *c*, for the simulated data. The class *c* is given in the plot titles. The *line* in the *middle of the box* shows the median, and the *bottom* and the *top of the box* show the 25th and 75th percentile, respectively. The whiskers extend to 1.5 times the inter-quartile range away from the *top* or *bottom of the box*, or to the furthest observations from the box. Data points outside the whiskers are plotted individually. We see that there is a large margin between the No bleeding class and large bleedings, and that there is a clear trend that the subspace distances correlate with the size of the bleeding

**Fig. 7:**
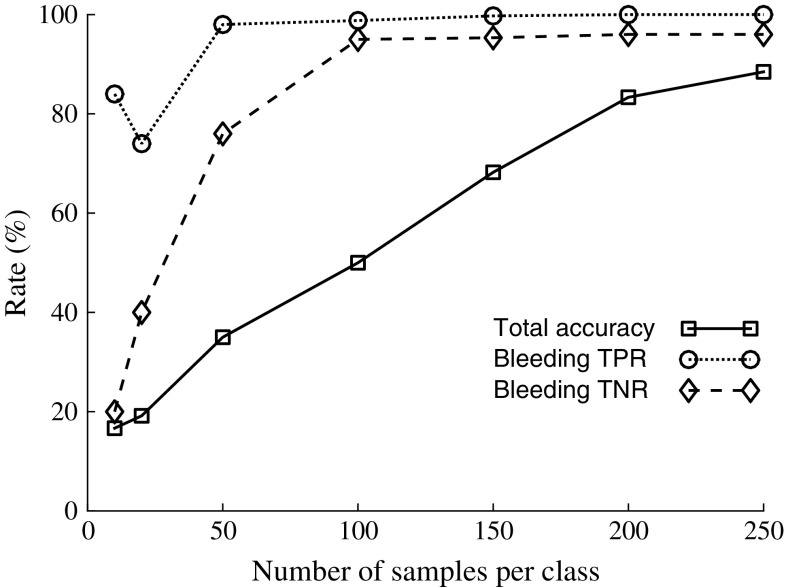
Rate of total classification accuracy for all classes, and true positive rate (TPR, same as sensitivity) and true negative rate (TNR, same as specificity) for bleeding, as a function of number of samples per class, for the simulated data. Total accuracy was calculated by summing all correctly classified observations (the main diagonal of the confusion matrix, see Table 2 that shows the result for 250 samples per class) and dividing by the total number of observations. To calculate TPR and TNR for bleeding, all bleeding sizes were combined into one class

The classification accuracy was overall high. Figure 5 shows the classification results for the laboratory measurements. The classification accuracy is 100 % for all classes. Table 2 and Figs. 6, 7 show the classification results for the simulated data. The classification accuracy is 82–96 % for all classes, i.e., when the full dataset was used with fivefold cross-validation. The true positive rate (sensitivity) for detection of bleeding (regardless of size) was 100 %, whereas the true negative rate (specificity) was 96 % (Fig. 7). However, a high accuracy required a relatively large training dataset. The classification accuracy dropped when smaller subsets of the data were used (Fig. 7). For both the laboratory and the simulated data, the bleeding size correlates with the subspace distances, i.e., the smaller the bleeding the closer it is to the No bleeding subspace (Figs. 5, 6). Bleedings of sizes close to each other are more similar to each other than sizes far apart. For the few misclassifications of simulated data, the predicted class is a bleeding with a nearby size (Table 2).

**Fig. 8:**
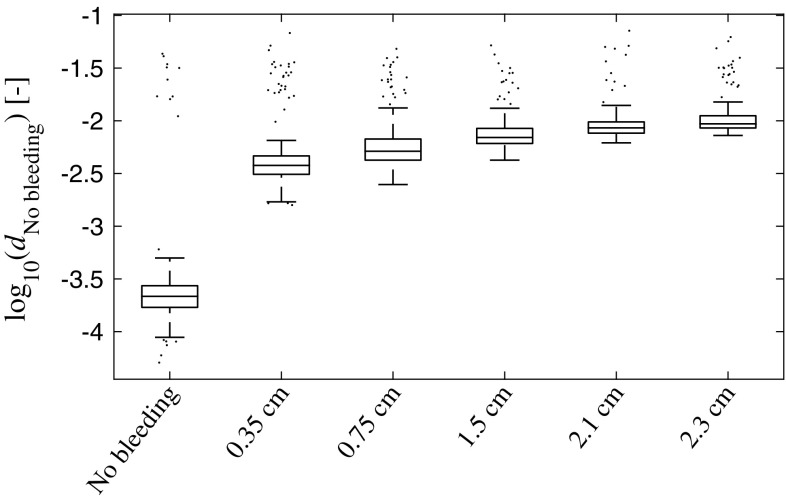
*Box plot* showing the distances to the No bleeding class of the training data, for all bleeding sizes of the simulated validation dataset. The *line* in the *middle of the box* shows the median, and the *bottom* and the *top of the box* show the 25th and 75th percentile, respectively. The whiskers extend to 1.5 times the inter-quartile range away from the *top* or *bottom of the box*, or to the furthest observations from the box. Data points outside the whiskers are plotted individually. We see that $d_{\mathrm {No\;bleeding}}$ correlates with bleeding size; which is consistent with the result for the original simulation dataset (Fig. 6)

**Table 2:** Confusion matrix for the classification result for the simulated data

	NB	0.2 cm	0.5 cm	1.0 cm	2.0 cm	2.5 cm
NB	240	5	4	0	0	1
0.2 cm	0	207	37	4	1	1
0.5 cm	0	33	205	9	0	3
1.0 cm	0	10	9	228	2	1
2.0 cm	0	5	5	1	228	11
2.5 cm	0	11	5	3	12	219

The rows show the actual class of a bleeding, and the columns show the predicted class. The number of observations correctly classified for each class is shown in the main diagonal

*NB* No bleeding

The outcome of the classification of the validation dataset based on training on the original simulation dataset is shown in Table 3 and Fig. 8. The classification accuracy is 99 % (Bleeding versus No bleeding), with only a few misclassifications (Table 3). Figure 8 shows that the size of the bleedings in the unseen dataset correlates with the subspace distance to the No bleeding class of the training data, i.e., smaller bleedings are closer to the No bleeding class.

**Table 3:** Confusion matrix for the classification result for the simulated validation dataset

	No bleeding	Bleeding
No bleeding	237	13
Bleeding	2	1248

The rows show the actual class, and the columns show the predicted class. The number of observations correctly classified for each class is shown in the main diagonal

**Fig. 9:**
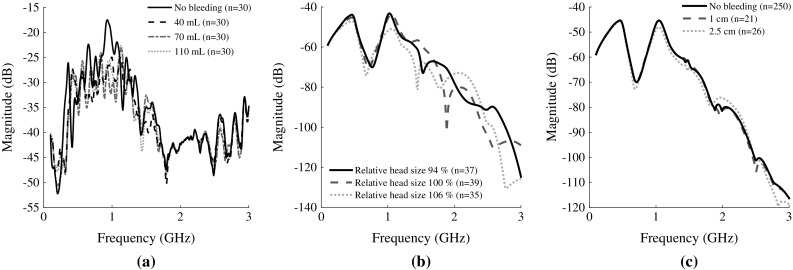
Mean magnitude for selected scattering parameters $S_{ij}$ for antennas positioned at opposite sides of the head close to the ears. Refer to Fig. 4, where the signal is transmitted between the two antennas situated at $y = 0$. The closest corresponding pair of antennas was chosen for the laboratory measurements. The direct signal pathway passes through the bleeding, see Figs. 2 and 4, where the depicted bleeding at position 1 was selected. The laboratory measurements (**a**) were lightly smoothed using Eilers’ algorithm [7] with $d = 2$ and $\lambda = 10$, to reduce noise and increase clarity. The large magnitude difference between laboratory and simulated data is most likely due to that signal is transmitted directly between antennas, without passing the head, to a larger degree for laboratory data. Furthermore, a simplified antenna model was used. **a** Laboratory data: no bleeding compared to the three different sizes of bleeding. **b** Simulated data: three different head sizes for simulations without bleeding. **c** Simulated data: no bleeding compared to two sizes of bleeding. Some bleeding sizes were omitted for increased clarity

Analysis of plots of raw reflection $S_{ii}$ and transmission coefficients $S_{ij}$ revealed that the effect of SDH is small compared to the effects of, e.g., head size, see Fig. 9. No consistent visual trends due to SDH were identified in plots of magnitude and phase for the measurements on the laboratory phantom. Even though plots of the mean magnitude indicate a difference (Fig. 9), the standard deviation for laboratory data is relatively large and obscures any possible trends due to SDH. For the simulated data, it was clear that head size is the parameter producing the largest variability, whereas differences due to bleeding size and position are relatively small (Fig. 9).

## Discussion

This study shows that MWT is a promising technology for prehospital detection of traumatic intracranial bleedings. The classifier distinguished all observations on three sizes of bleedings and no bleeding for the laboratory SDH model (Fig. 5). Furthermore, using a finite element model, we simulated a large number of bleedings with different sizes, at various positions, in crania of different sizes. The classifier identified SDH with high accuracy (Table 2; Fig. 7 ), despite the relatively high variability from varying head size (Fig. 9b, c). Furthermore, classification accuracy was high on an unseen dataset with different values of head and bleeding sizes (Table 3). This indicates that the model was not overfitted to the original dataset.

This conceptual study evaluates the hypothesis that it is possible to detect small signal changes caused by intracranial bleedings in a heterogeneous dataset with high variability, due to inter-individual differences between patients. Results for the phantoms constructed in the human cranium indicate that it is possible to detect SDH using the current microwave system in conjunction with the classifier, with consideration taken to the variability caused by taking the helmet on and off (Fig. 5). However, this does not consider the inter-patient variability. It was not feasible to construct physical phantoms of many different head sizes. Therefore, the simulations were performed to evaluate the impact of inter-patient variability. The results indicate that it is possible to differentiate bleeding sizes (Fig. 6), if a large training dataset is available (Fig. 7).

Bleeding size correlated with the subspace distance to the No bleeding class and bleedings of nearby sizes were more similar to each other compared with bleeding sizes far apart (Figs. 5, 6, 8). We have previously shown that there is good potential for estimating the position of a bleeding [5]. These results combined point to that MWT, in addition to reliable SDH detection, can be of further clinical value by estimating size and position of the lesion. In particular, the potential for size estimation can be valuable for monitoring patients, to detect an expanding bleeding before the patient’s condition deteriorate.

The laboratory model of SDH was made in a human cranium. This was important to make the model more realistic, especially since it was expected that the cranium would attenuate the signal substantially [32]. The SDH model was simplified due to practical reasons. It was not feasible to construct an advanced phantom with a realistic anatomy of the human brain. However, the healthy human brain is symmetric with respect to the left and right hemispheres. Our hypothesis is that the altered scattering pattern of the microwave signals, due to the asymmetry introduced by a lesion, is the main reason for that bleedings can be detected by the classifier. In this regard, the model is suitable. A disadvantage with the laboratory phantom used in this study is that although the bleeding compartments were cut out and filled with bleeding solution in a meticulous manner, it cannot be guaranteed that small pockets of air are not present. Furthermore, the dielectric properties could not be matched closely to published values across the full frequency interval (Fig. 1). In future studies, it would be valuable to confirm the results obtained in this study using improved and more realistic models of the brain, such as [21].

The laboratory data showed relatively large variability/noise (Fig. 9). This was to a large degree caused by taking the helmet on and off. It was done to mimic the clinical scenario, where the helmet position will vary. From Fig. 5, we see that the variability from taking the helmet on and off manifests in the subspace distance. The repeated observations in groups of three are close together, while the difference between the groups is considerably larger. The differences between the SDH models were larger than the differences due to helmet position (Fig. 5). The classification accuracy was high, and the trends for the subspace distances for different classes followed the numerical simulations (Figs. 5, 6). Thus, the classifier seems capable of identifying the pattern due to bleeding, despite that this effect was small relative to other sources of variability/noise, and the apparent strong cross talk between antennas (Fig. 9). This was achieved for a relatively small dataset, as compared to the numerical data where a large training dataset was necessary (Fig. 7). A plausible explanation is that for the laboratory phantom the cranium size and bleeding position were fixed and the effect of bleeding size should then be more readily detectable.

For the numerical simulations, a 2D electromagnetic finite element method was employed to achieve a fast and efficient simulation tool, which described the physics for the measurement setup reasonably well. The model was rather simple for practical reasons. Our aim was not to create simulation data that are highly realistic with respect to patient anatomy, but to create a realistic model of the variability due to head size, bleeding position and bleeding size, and CSF, as will be experienced for future measurements on patients.

We consider this to be a suitable approach to simulate patient measurements in the context of a classification test, since the fundamental physics and numerical method are well known and understood. The main limitations of the simulation model are that the anatomy was simplified and that simulations were performed in 2D, which limits the antenna structures that are possible to realize.

Accurate detection may require that the classification algorithm is trained on a relatively large number of patients (Fig. 7). Further development of the classification method, to realize a high classification accuracy for smaller training datasets, may decrease the number of patients needed in future clinical studies.

## Conclusion

This study presents a new method for detection of traumatic intracranial bleedings using MWT in conjunction with a diagnostic algorithm. Through laboratory measurements and numerical simulations, we show that the method has potential for accurate detection of clinically significant bleedings. This is promising for the development of a simple-to-use instrument to recognize patients with occult, severe brain injury already in the prehospital setting.
